# Observations from a prospective small cohort study suggest that CGRP genes contribute to acute posttraumatic headache burden after concussion

**DOI:** 10.3389/fneur.2022.947524

**Published:** 2022-08-05

**Authors:** Michael F. La Fountaine, Asante N. Hohn, Caroline L. Leahy, Joseph P. Weir, Anthony J. Testa

**Affiliations:** ^1^Department of Physical Therapy, School of Health and Medical Sciences, Seton Hall University, Nutley, NJ, United States; ^2^Departments of Medical Sciences and Neurology, Hackensack Meridian School of Medicine, Nutley, NJ, United States; ^3^Department of Health, Sport and Exercise Sciences, University of Kansas, Lawrence, KS, United States; ^4^Osness Human Performance Laboratories, University of Kansas, Lawrence, KS, United States; ^5^Center for Sports Medicine, Seton Hall University, South Orange, NJ, United States

**Keywords:** mild traumatic brain injury, acute post-traumatic headache, calcitonin gene-related peptide, post-concussion syndrome, genotyping

## Abstract

**Introduction:**

Post-traumatic headache (PTH) is commonly reported after concussion. Calcitonin gene-related peptide (CGRP) is implicated in the pathogenesis of migraine. We explored how single nucleotide polymorphisms (SNPs) from CGRP-alpha (CALCA) and the receptor activity modifying protein-1 (RAMP1) related to headache burden during the first week after concussion.

**Methods:**

A prospective study was performed in 34 collegiate athletes who sustained a concussion. Participants completed the symptom evaluation checklist from the SCAT3 within 48 h of injury (V1), and again 4 (V2) and 7 (V3) days after injury. For each visit, the self-reported score (0–6) for headache, pressure in head, blurred vision, and sensitivity to light/noise were reported and summed to calculate the headache burden. A saliva sample was obtained and genotyped for CALCA (rs3781719) and RAMP1 (rs10185142). RAMP1 (TT, TC, CC) and CALCA (AA, AG, GG) were dichotomized (A+, A- and T+, T-, respectively), and concatenated (T+A+, T+A-, T-A+, T-A-) for analyses.

**Results:**

Headache Burden at Visit 1 was greatest in T+A+ compared to T-A+, and trended toward a significant difference with T+A-. Repeated-measures ANOVA revealed the presence of significant visit main effects (*p* < 0.001, η^2^ = 0.404), but the group (*p* = 0.055) and interaction effects only trended (*p* = 0.094). Pearson's χ^2^-tests revealed that 88% of those with return-to play (RTP) exclusions ≥15 days had PTH with multi-sensory symptoms (PTH+SENS) as compared to 35% in those with RTP < 14 day.

**Conclusion:**

Knowledge of RAMP1 and CALCA genotypes appear to improve an understanding the presenting features and magnitude of headache burden after concussion injury.

## Introduction

Posttraumatic headache (PTH) is classified by the International Classification of Headache Disorders (ICHD)-3 as a secondary headache disorder (1). It is among the most commonly reported presenting symptoms after concussion and it is unpredictable and difficult to manage with potentially disabling impacts on the sufferer (2–5). PTH can be defined as “acute PTH” if the onset of headache is within 7 days of injury and resolves within 3 months, or “persistent PTH” if remission is not achieved during the “acute” window (1, 6). Unsurprisingly, PTH is also one of the most prominent and frequently reported symptoms among those persons with post-concussion and persistent post-concussion syndromes (PCS and PPCS, respectively) (7), which are an atypical resolution of concussion symptoms that can extend for several months, and in rare cases years beyond the initial injury (8–10). The cumulative incidence of PTH was found to be ~91% during the first year following mild traumatic brain injury (MTBI) (11). A recent review article highlighted that numerous pre- and post-injury factors interact and contribute to the presentation of PTH (12). The opportunity to intervene and remediate the impact and occurrence of PTH requires a comprehensive post-injury evaluation and the development of an individualized plan of care to account for co-presenting and persisting symptoms. While there are no clinical practice guidelines available to drive clinical decisions on the treatment of PTH after concussion, numerous independent agencies such as the American Migraine Foundation, the American Headache Society and healthcare networks with public-facing resources including but not limited to the Healthcare Outcomes Performance Company, the Children's Hospitals of New Orleans and Orange County, respectively, offer guidance on self-care strategies, termed “headache hygiene” to manage headache symptoms by establishing daily routines, avoiding known triggers, stress management, and engaging in physical activity, among other behaviors.

Although its exact etiology is undefined, PTH severity and presenting features are likely related to pre-morbid conditions including the experience of prior migraine, a family history of migraine (13, 14) and its characteristics are suggested to resemble migraine or tension-type headaches (12). In the briefest of explanations, a migraine headache involves activation and sensitization of first-order trigeminovascular neurons, which creates a cascading effect through the release of vasoactive- and neuropeptides and local inflammatory factors; these in turn, sensitize and activate structures linked through second- and third-order neurons that culminate in the perception of pain and other abnormal sensations during the headache episode (15–17). One of the key modulators of this cascade is the neuropeptide calcitonin gene-related peptide (CGRP) which has distributions in the central and peripheral nervous systems (18–21). CGRP has 2 isoforms (i.e., α and β, respectively) that are expressed from the calcitonin related polypeptide α and β genes (i.e., CALCA and CALCB, respectively), with CALCA being the predominant form of CGRP expressed in the trigeminal ganglia (22, 23). The CGRP receptor has three subunits including the receptor-activity modifying protein 1 (RAMP1) which modulates rate limiting functions of the receptor (24) and provides specificity for ligand binding (25). RAMP1 overexpression has been demonstrated to cause light-aversive behavior and central CGRP-induced allodynia in transgenic mice models (26, 27). CGRP synthesis is not fully understood but has been shown to be upregulated in models of nerve damage whereby the inflammatory response to traumatized tissues increases its synthesis and expression (28, 29). CGRP is emerging as an important target for pharmacological treatments that prevent migraine from happening or are used when an attack occurs (30).

When a migraine phenotyping approach was applied to the characterization of PTH in youths after concussion, those with concurrent migraine-like symptoms (i.e., multi-sensory symptoms) were more likely to experience persistent symptoms compared to those who did not have PTH or had PTH without migraine-like symptoms (31). PTH symptoms are thought to have a hereditary component in an undefined percentage of cases and is most often explored *via* interview to ascertain migraine history or through the use of genotyping. An individual's personal history of having a migraine headache was associated with an increased risk of experiencing migraine-like PTH (32) and individuals who sustained a concussion that has a positive family history of migraine headaches were also found to be more likely to present with migraine-like PTH (33). With such a limited understanding of the mechanisms that contribute to PTH after concussion, the opportunity exists to apply contemporary knowledge of migraine mechanisms to improve our understanding of PTH and perhaps identify targets for potential intervention. Improvements in genotyping technologies and services have made it possible to perform association studies that explore how genotypic variation(s) in DNA base pairs from adenine (A), cytosine (C), guanine (G), and thymine (T) coalescence in candidate single nucleotide polymorphisms (SNP) and pathways to underly the presentation of symptoms and/or impart risk for future morbidities. Therefore, the purpose of this investigations was to determine whether single nucleotide polymorphisms (SNP) from CALCA and RAMP1 in the CGRP pathway play a role in the presentation of headache symptom burden as quantified by the Sport Concussion Assessment Tool (SCAT) during the first week after concussion among college-aged athletes.

## Materials and methods

### Participants

The study procedures were approved by the institutional review board and all participants provided written informed consent prior to participating. As part of the clinical evaluation at the time of a suspected injury that occurred during practice or competition, the athlete(s) were evaluated and diagnosed by the sports medicine staff using accepted clinical practices guidelines of concussion assessment for both immediate on-field and subsequent office-based follow-up evaluations that included symptom evaluation (34, 35). To determine the presence of a concussion, an injured athlete underwent a physical and neurological examination, and an assessment of cognitive function and standing balance. These evaluations were aided by the use of the SCAT, which is an industry standard in the evaluation of a suspected concussive head trauma (34–36). If the injured party had positive signs and symptoms that were consistent with concussion, they were withheld from participation. In the event of a medical emergency (i.e., Glasgow Coma Scale <13, prolonged loss of consciousness, or worsening somatic/cognitive or other neurological symptoms), the injured athlete was taken to the emergency department. Otherwise, the injured party was re-evaluated after a brief rest period to confirm the presence of symptoms. The final diagnosis of concussion was made by a physician trained in the care of MTBI. Once the diagnosis was confirmed, the injured athlete was monitored by sports medicine staff on a consistent daily basis over the subsequent days until the symptoms resolved. Clinical decision making on return-to-play (RTP) of concussed athletes was made by the sports medicine staff and did not include any input from data collected in the research study. The number of days that an individual with concussion was symptomatic was tracked clinically and for this study were characterized as having symptoms for <14 days (e.g., RTP < 14 days) or more than 15 days (e.g., RTP ≥ 15 days). Once the concussed athlete was free of symptoms, they began a 5-stage clinically-driven RTP protocol.

For the purposes of enrollment in the prospective observational study, a potential participant with a concussion was deemed by the principal investigator to have capacity to provide informed consent by demonstrating the ability to orient to person, place, time and a current event. To be eligible for the research study, the athlete with a diagnosed concussion injury must have sustained their injury within the previous 48 h, be able to demonstrate capacity to provide written informed consent, not be taking medications with direct or indirect actions on the cardiovascular or central nervous systems, and be free of acute illness or trauma that would otherwise minimize their ability complete the study procedures. The number of diagnosed concussions that each participant sustained during their lifetime was obtained *via* self-report.

### Data collection

The initial study observation occurred in a research laboratory within 48 h of injury (e.g., Visit 1) and was repeated 4 and 7 days later (e.g., Visits 2 and 3, respectively). Data collection occurred between 8:00 a.m. and 12:30 p.m. for all study visits and the subsequent study visits occurred within a ± 30-min window of the start time from the initial visit to mitigate the impact of daily activities on symptom presentation. Participants were required to abstain from consuming alcohol-containing or caffeinated beverages for at least 12 h prior to data collection. The laboratory environment was thermoneutral (i.e., ~21°C) and overhead lights were turned off; ambient lighting in the room came from exterior windows.

On the first visit, participants were given a saliva collection vial (Oragene Discover, OGR-500, DNA Genotek, Ottawa, CA) to provide a specimen that would then be analyzed by a commercial laboratory (DNA Genotek, Ottawa, CA). Each participant was instructed to gather saliva in their mouths and then spit/drool into the collection vial. Once a sufficient sample was obtained (~2 mL of saliva without bubbles), the participant was instructed to firmly close the lid which then released a stabilizing liquid into the vial. The study team member then unscrewed the collection funnel, placed a cap and then inverted the collection vial several times for 5 s to mix the saliva and stabilizing liquid. The specimens were stored in a laboratory freezer (So-Low Ultra-low Chest Freezers; Laboratory Supply Network, Inc, Atkinson, NH, USA) at −80°C until they were shipped to the laboratory for processing. For processing at the laboratory (DNA Genotek, Ottawa, CA), DNA extraction was completed using 300 uL of saliva as input on a KingFisher Flex (Thermofisher) automated extraction instrument with a Pro-K pre-treatment. Following extraction, all samples were run through a Quant-iT PicoGreen dsDNA quality check to ensure that the yield was sufficient to achieve a 5 ng/uL concentration threshold that is required to load on the DTCv2 array. Isolated DNA was quantified using Quant-iT PicoGreen dsDNA assay kits (Thermofisher) and was qualitatively assessed using a spectrophotometer. DNA was then processed on a customized version of the Precision Medicine Research genotyping array (Thermofisher) according to the manufacturer's protocols.

A specialized software program written in Python (version 3.9.1, www.python.org) was used to locate the candidate SNPs by referencing the rsID number within each participant's report and to create an output with the respective genotypes for the targeted SNPs. For the purposes of this investigation, SNPs for RAMP1 (rs10185142; TT, TC, CC) and CALCA (rs13781719; AA, AG, GG) were extracted from the report for analyses. These were the only SNPs for CGRP, CALCA and RAMP1 available in the report of ~500 k SNPs that were identified by the laboratory and is a noted limitation of this study. The genotyped data were then imported to a workbook where participant data and outcomes were collated for subsequent processing and statistical analyses. To facilitate statistical analyses, RAMP1 (TT, TC, CC) and CALCA (AA, AG, GG) genotypes were dichotomized (T+, T- and A+, A-, respectively), and concatenated (T+A+, T+A-, T-A+, T-A-).

On each of the three study visits, participants completed the SCAT3 by rating their current experience (scale 0–6) for the 22 listed symptoms. For this analysis, the self-reported scores for headache, pressure in head, blurred vision, sensitivity to light, and sensitivity to noise were summed to generate a total “headache burden” score for each participant's visits. Participant responses for Visit 1 were then categorized as reporting no PTH symptoms (NoPTH), PTH symptoms (PTH+), PTH with multi-sensory symptoms (PTH+SENS). For these categorizations, participants were defined using their self-reported SCAT3 rating as having: “NoPTH” if headache and pressure in head symptoms were 0; “PTH+” if headache and pressure in head symptoms were ≥1 and blurred vision, sensitivity to light and sensitivity to noise were each 0; and, “PTH+SENS” if headache and pressure in head symptoms were ≥1 and any single report or combination of blurred vision, sensitivity to light and sensitivity to noise were ≥1. This categorical approach to expound the headache milieu was adapted from the headache phenotyping model described by Kamins et al. whose investigation used the Post-concussion Symptom Inventory (37) to examine non-migraine vs. migraine-like headache characteristics in youths with PCS and PTH (31).

### Statistical analysis

Values are expressed as group mean ± SD unless otherwise indicated. Statistical analyses were performed without the T-A- genoset included because there was only 1 person in the cohort; this participant was also the only individual to report having no headache symptoms at Visit 1. Analysis of variance (ANOVA) was performed to identify group differences (i.e., T+A+, T+A-, T-A+) for Visit 1 headache burden. Repeated measures ANOVA were performed to explore group (T+A+, T+A-, T-A+) differences in headache burden across the study visits (i.e., Visit 1, Visit 2, Visit 3). The nature of significant group main effects was explored with Tukey *post-hoc* tests. Pearson's χ^2^ tests were performed to understand how categorical assignment of PTH characteristics (NoPTH, PTH+, PTH+SENS), duration of time until participants entered the RTP protocol (RTP ≥ 15 days, RTP <14 days) and CALCA+RAMP1 subgroup distributions were related. Statistical analyses were completed using IBM SPSS Statistics 26 (IBM, Armonk, NY, USA) and figures were created with GraphPad Prism (version 8.4 for Windows, GraphPad Software, San Diego, CA, USA). An *a priori* level of significance was set at *p* ≤ 0.05.

## Results

**Figure 1:**
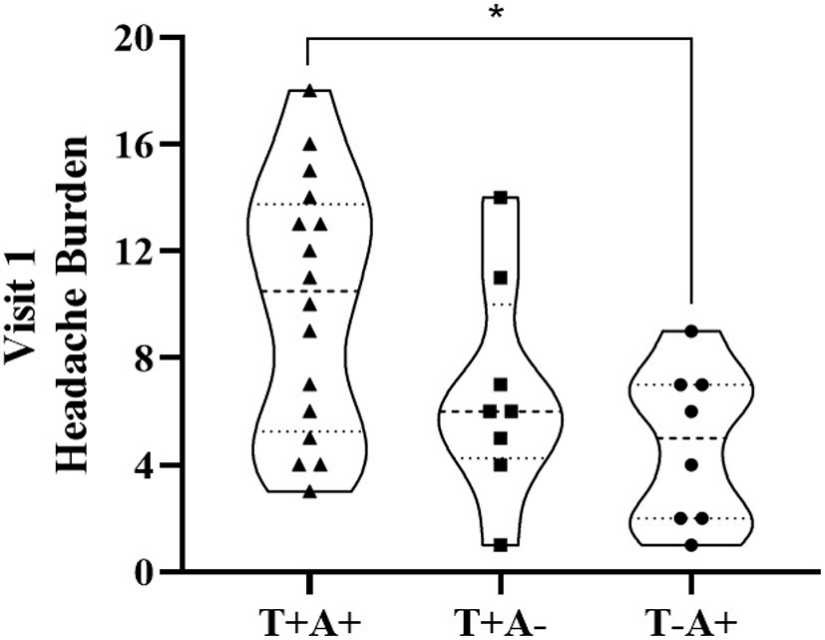
Truncated Violin plot demonstrating Headache Burden at Visit 1 among RAMP1 + CALCA genoset groups. Each violin plot presents the group median (- - - -) and interquartile range (·····). The truncation of plots represents the minimum and maximum observation for each group. The dark marks within each plot represent individual participant data. *p < 0.05.

**Figure 2:**
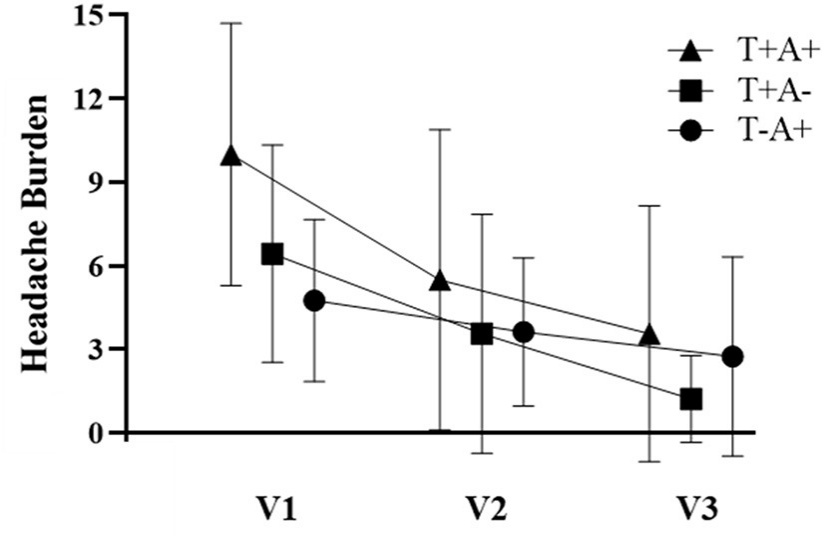
Headache Burden for RAMP1 + CALCA genotype subgroups across the 3 study visits (V1, V2, and V3, respectively). Data are presented as group mean ± SD. Repeated-measures ANOVA revealed the presence of significant group (*p* < 0.001, partial η^2^ = 0.633) and visit main effects (*p* < 0.001, η^2^ = 0.404), but the interaction effect did not achieve statistical significance (*p* = 0.125).

**Figure 3:**
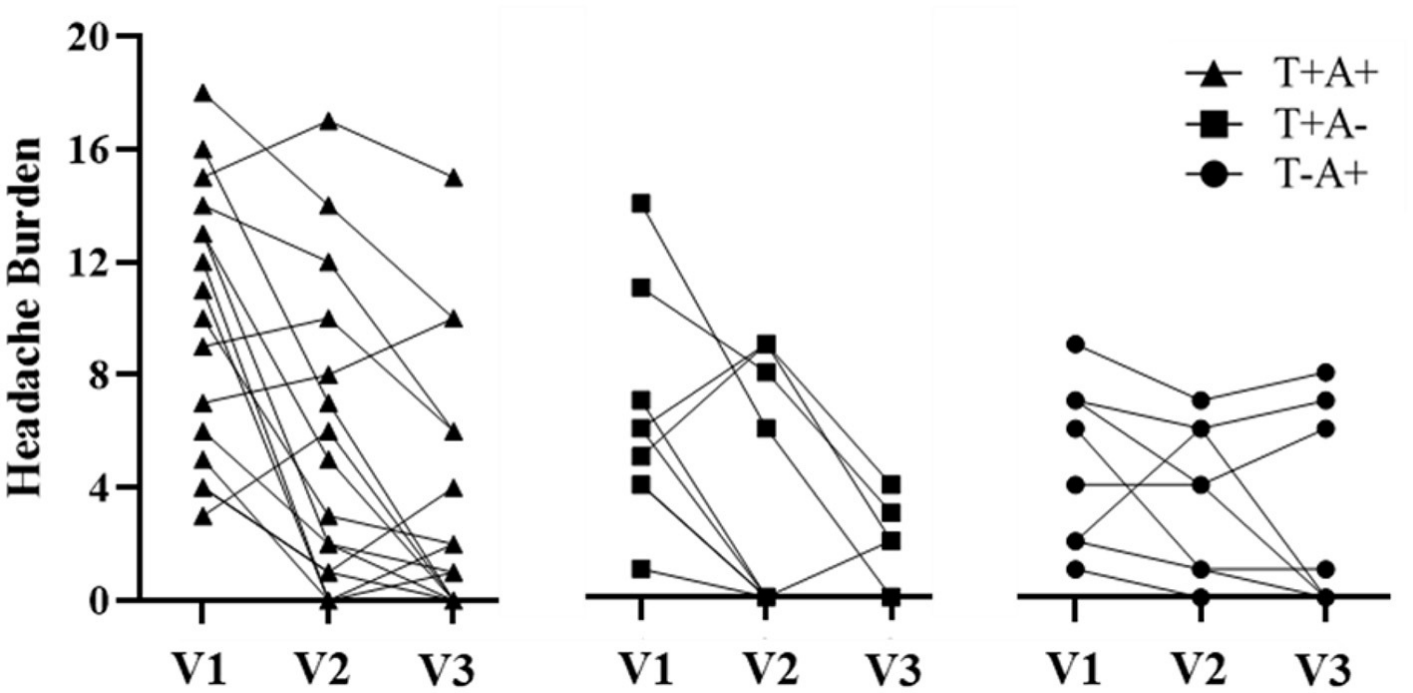
Headache Burden among individual participant data within RAMP1 + CALCA genotype sub-groups across study visits (V1, V2, and V3, respectively).

Participant demographics are provided in Table 1 for the 34 participants who enrolled in the study. Self-reported symptoms scores for each of the 5 items are provided as group mean ± SD for each of the three study visits are provided in Table 2. The number of participants for the RAMP1 + CALCA genotype sub-groups is as follows: T+A+ (*n* = 16), T+A- (*n* = 9), T-A+ (*n* = 8), T-A- (*n* = 1). Headache Burden at Visit 1 is presented as a truncated violin plot for the RAMP1 + CALCA genotype sub-groups (Figure 1). ANOVA revealed that the omnibus model was significant (*p* = 0.015, partial η^2^ = 0.245). Mean pairwise comparisons between groups using the Tukey HSD showed sig greater headache scores between T+A+ and T-A+ (Tukey adjusted *p* = 0.018) but NS differences between: 1) T+A+ vs. T+A- (Tukey adj *p* = 0.17) and 2) T+A- vs. T-A+ (Tukey adj *p* = 0.68). However, the unadjusted 95% CI of the mean differences for T+A+ vs. T+A- (95% CI of mean difference = 0.02–7.1) and for T+A+ vs. T-A+ (95% CI = 1.6–8.9) both excluded zero. Repeated-measures ANOVA revealed the presence of significant group (*p* < 0.001, partial η^2^ = 0.633) and visit main effects (*p* < 0.001, η^2^ = 0.404), but the interaction effect did not achieve statistical significance (*p* = 0.125; Figure 2 as groups, and Figure 3 as individual data within RAMP1 + CALCA genotype sub-groups). No participant demographics or past concussion history were found to be contributors to the magnitude of headache symptoms.

**Table 1:** Participant demographics.

***n***	**34**
Age (yrs)	19.7 ± 1.4
Height (m)	1.75 ± 0.12
Weight (kg)	73.2 ± 14.1
BMI (kg/m^2^)	23.8 ± 3.4
Prior concussions	1.3 ± 1.5
Gender (F/M)	23/11
**Ethnicity**
AA/Cauc/Hisp (*n*)	4/27/3

Data are presented as group mean ± SD. AA, African American; BMI, body mass index; Cauc, Caucasian; F, female; Hisp, Hispanic; M, male.

**Table 2:** Self-reported symptom scores from SCAT3.

	**48 h**	**4 days**	**7 days**
Headache	2.4 ± 1.4	1.5 ± 1.4	0.9 ± 1.1
Pressure in head	2.1 ± 1.5	1.3 ± 1.2	0.7 ± 0.9
Blurred vision	0.5 ± 0.9	0.3 ± 0.8	0.3 ± 0.8
Sensitivity to light	1.8 ± 1.6	1.1 ± 1.3	0.6 ± 1.0
Sensitivity to noise	1.3 ± 1.5	0.6 ± 1.1	0.3 ± 0.7
Total headache burden	8.0 ± 4.9	4.4 ± 4.5	2.6 ± 3.8

*Data are presented as group mean ± SD*.

The study sample had an equal number of participants based on when they were cleared by clinicians to enter the RTP protocol [i.e., RTP < 14 days (*n* = 17); RTP ≥ 15 days (*n* = 17), respectively]. During the study evaluation at Visit 1, 3 participants (9%) reported having NoPTH, 10 (29%) had PTH+ and 21 (62%) had PTH+SENS. Pearson's χ^2^ tests revealed that 88% (*n* = 15) of those with RTP ≥ 15 days had PTH+SENS as compared to 35% (*n* = 6) in those with RTP < 14 days [χ^2^ (2, *N* = 34) = 10.5, *p* = 0.005; Table A in e-Appendix 1]. More specific to the RAMP1 + CALCA subgroup contributions, Pearson's χ^2^ tests revealed that 57% (*n* = 12) with PTH+SENS had a T+A+ genoset while a further 29% (*n* = 6) with PTH+SENS had a T+A- genoset [χ^2^ (4, *N* = 34) = 16.2, *p* = 0.013; Table B in e-Appendix 1]. In an exploratory analysis, it was found that 86% (*n* = 18) of those who reported PTH+SENS had a T+ for RAMP1 [χ^2^ (2, *N* = 33) = 5.8, *p* = 0.055; Table C in e-Appendix 1].

## Discussion

The current analyses provide a proof-of-concept to suggest that the combined T+A+ genoset from RAMP1 (rs10185142) and CALCA (rs13781719) is associated with a greater headache burden early after concussion injury when compared to other genosets from the SNPs used in this study, albeit the rates of recovery appear to be similar. The data also suggests that the presence of a T allele for the RAMP1 SNP had a significant effect on the presentation of multisensory symptoms with PTH, which is mechanistically supported by transgenic mice models where RAMP1 overexpression caused light-aversive behavior and central CGRP-induced allodynia (26, 27). Our observations came from a novel adaptation of the widely used SCAT symptom survey which aided in the identification of divergent PTH sequalae such that those with PTH and multisensory symptoms (i.e., blurred vision, sensitivity to light and sensitivity to noise) at Visit 1 within 48 h of injury were two and three times more likely, respectively, than those with PTH and no multisensory symptoms or No PTH to be excluded from participation for more than 15 days after injury (i.e., RTP ≥ 15 days). The use of a migraine phenotyping approach to characterize PTH in youths (31) appears to provide meaningful insight to the PTH sequelae in college-aged athletes (i.e., young adults) using the SCAT following concussion injury.

CGRP is emerging as an important target for pharmacological treatments that prevent migraine from happening or are used when an attack occurs. The class of medications known as CGRP-receptor antagonists (i.e., gepants) are often recommended to patients that have not responded favorably to other medications (i.e., triptans), after adverse reaction or they are contraindicated (i.e., patients with cardiovascular-related issues) (30). These medications work by blocking the CGRP receptor by directly competing for the binding site of the endogenous ligand CGRP, and therefore, inhibiting the physiological and cellular effects of CGRP (29). CGRP receptor antagonists have known hepatotoxic side effects and anti-CGRP monoclonal antibodies (anti-CGRP mAbs) are available as an alternative option (38). These medications have a high target specificity which reduces unintended side effects (39) while providing efficacy in preventing episodic and chronic migraine, and reducing the number and severity of headache days (38).

There are limited studies available to demonstrate the use of CGRP-related therapies in humans after concussion injury. In one open label safety/efficacy trial with erenumab (in 100 patients with PTH attributed to MTBI), it was found that 12 weeks of treatment reduced the number of headache days in patients by an average of 2.8 days with only 2 participants discontinuing treatment due to an adverse event (40). In a retrospective chart review of 3 patients with PTH following concussion, all individuals reported having an improvement in their headache symptoms (2 received erenumab, 1 received fremanezumab) without experiencing adverse side effects (41). In a second retrospective chart review of concussed patients with a PTH migraine phenotype, a preliminary analysis found that 3 anti-CGRP mAbs (erenumab, fremanezumab, galcanezumab) reduced headache severity and frequency while also decreasing the number of concussion symptoms (42). Authors in this final report indicated that subset of patients had a more robust response to treatments and that switching medications was beneficial in some cases. It is unclear if any participants from these studies were genotyped and all participants would have been characterized as having persistent PTH.

Despite the lack of empirical evidence demonstrating the use of CGRP agents in humans, there are several compelling experimental demonstrations in animal models that reinforce the proposed role of CGRP on PTH after head injury. In an MTBI model of PTH that used male mice, it was found that “sequestration” of CGRP prevented both acute and persistent PTH, while delaying an anti-CGRP monoclonal antibody treatment until central sensitization was achieved, proved to be ineffective in preventing persistent PTH (43). The authors concluded that “…*the mechanisms involving CGRP underlie the expression of acute PTH, and drive the development of central sensitization, increasing vulnerability to headache triggers and promoting persistent PTH*” (43). In a separate study of PTH after MTBI that used female Sprague Dawley rats, the authors sought to evaluate if the purported increase in the susceptibility of females to experience PTH after concussion was related to CGRP (44). The authors found that: (1) PTH behaviors and the responsiveness to anti-CGRP monoclonal antibody treatment is different between the sexes; (2) female rats displayed a prolonged period of cephalic hyperalgesia (i.e., increased sensitivity to pain in the head); (3) increased responsiveness to a headache trigger; and, (4) a poorer effectiveness of an early and prolonged anti-CGRP treatment (44). Unlike the study in male mice, the authors of the female rat study concluded that “…*the increased risk of females to develop PTH may be linked to enhanced responsiveness of peripheral and/or central pain pathways and a mechanism independent of peripheral CGRP signaling*” (44). Unfortunately, our study did not have a sufficient sample size or equal numbers of males and females to examine the impact of sex on PTH characteristics, let alone across the RAMP1+CALCA genoset. However, females represented 2/3rd of our study cohort and the omnibus statistical model included gender as a covariate so we cannot rule out the effect.

There are several limitations to consider when placing the current findings in context. The small sample sizes of this cohort study are acknowledged and speculation about physiological or functional differences between the genotype subgroups are not being made. A larger and more ethnically diverse population of participants with acute concussion injury should be performed using a comprehensive battery of validated headache symptoms surveys with longitudinal follow-up and appropriate outcome measurements. The T-allele for RAMP1 (rs10185142) and A-allele for CALCA (rs13781719) used in this study are likely to be part of a larger genoset with an undefined number of other SNPs or loci from the CGRP pathway that contribute to the increased presentation of headache symptoms after concussion injury. We do not contend that these specific CGRP SNPs are the only ones that could produce similar findings given that another study to explore the role of CALCA and RAMP1 genes on migraine found no significant associations of CALCA or RAMP1 on their headaches; albeit with different SNPs for CALCA (rs3781719, rs145837941) and RAMP1 (rs3754701, rs7590387) used in their analyses (45). In addition, the association study was performed in adults from a population of adults who resided in southeastern Australia and were predominately of European descent. With this being said, the pathogenesis of migraine headaches and PTH do have fundamental differences that may have contributed to a presumed link between CALCA gene and RAMP1 locus in the data presented herein, but not in the study of adult migraineurs.

## Conclusion

The current study represents a novel demonstration of the potential impact of how genetic variability among genes that are associated with the CGRP pathway seemingly contributes to the presentation of acute PTH, and more specifically, PTH with multi-sensory symptoms in college-aged athletes after concussion injury. These data also suggest that the duration of time that an individual may experience symptoms after concussion could be related to the RAMP1 and CALCA genes. Adaptation of the SCAT3 instrument to stratify the PTH experience after concussion appeared to identify a divergent sequela related to headache experiences after injury. The use of patient genotyping alongside comprehensive interpretation of self-reported symptoms early after concussion could become an invaluable clinical tool to identify those individuals who may be susceptible to a greater symptom burden, as well as to aid in the development of an objective prognosis. Similarly, these data provide a compelling mechanistic basis for the use of CGRP-related therapies after concussion.

## Data availability statement

The data are not publicly available due to the on-going nature of the study analyses. Requests to access the datasets should be directed to lafounmi@shu.edu.

## Ethics statement

The studies involving human participants were reviewed and approved by Hackensack Meridian Health Institutional Review Board. The patients/participants provided their written informed consent to participate in this study.

## Author contributions

ML: conceptualization and methodology, formal analysis, investigation, supervision, project administration, funding acquisition, writing of original draft, and final draft approval. JW: formal analysis, writing of original draft, and final draft approval. AH and CL: investigation and final draft approval. AT: investigation, supervision, and final draft approval. All authors contributed to the article and approved the submitted version.

## Funding

This research was supported by the New Jersey Commission for Brain Injury Research-Individual Research Grant #CBIR16IRG025.

## Conflict of interest

The authors declare that the research was conducted in the absence of any commercial or financial relationships that could be construed as a potential conflict of interest.

## Publisher's note

All claims expressed in this article are solely those of the authors and do not necessarily represent those of their affiliated organizations, or those of the publisher, the editors and the reviewers. Any product that may be evaluated in this article, or claim that may be made by its manufacturer, is not guaranteed or endorsed by the publisher.
