# Supplementary material for: Observations from a prospective small cohort study suggest that CGRP genes contribute to acute posttraumatic headache burden after concussion
Source: Front Neurol. 2022 Aug 5;13:947524. doi: 10.3389/fneur.2022.947524 (PMC9389220; doi:10.3389/fneur.2022.947524)
Supplement: Supplementary file 1 [file Table_1.DOCX]

|  |  |  |  |  |  |  |
| --- | --- | --- | --- | --- | --- | --- |
|  | Table A. | No PTH | PTH+ | PTH+SENS | Total |  |
|  | RTP <14 days | 3 | 8 | 6 | 17 |  |
|  | RTP ≥ 15 days | 0 | 2 | 15 | 17 |  |
|  | Total | 3 | 10 | 21 | 34 |  |
|  |  |  |  |  |  |  |
|  | Table B. | No PTH | PTH+ | PTH+SENS | Total |  |
|  | T-A- | 1 | 0 | 0 | 1 |  |
|  | T-A+ | 0 | 5 | 3 | 8 |  |
|  | T+A- | 1 | 2 | 6 | 9 |  |
|  | T+A+ | 1 | 3 | 12 | 16 |  |
|  | Total | 2 | 10 | 21 | 34 |  |
|  |  |  |  |  |  |  |
|  | Table C. | No PTH | PTH+ | PTH+SENS | Total |  |
|  | RAMP1 T- | 0 | 5 | 3 | 8 |  |
|  | RAMP1 T+ | 3 | 5 | 18 | 26 |  |
|  | Total | 3 | 10 | 21 | 34 |  |
|  |  |  |  |  |  |  |
